# Stable diffusion gradients in microfluidic conduits bounded by fluid walls

**DOI:** 10.1038/s41378-024-00698-1

**Published:** 2024-06-20

**Authors:** Federico Nebuloni, Cyril Deroy, Peter R. Cook, Edmond J. Walsh

**Affiliations:** 1 Department of Engineering Science, Osney Thermo-Fluids Institute, University of Oxford, Oxford, OX2 0ES UK; 2 Sir William Dunn School of Pathology, University of Oxford, Oxford, OX1 3RE UK

**Keywords:** Engineering, Physics

## Abstract

Assays mimicking in vitro the concentration gradients triggering biological responses like those involved in fighting infections and blood clotting are essential for biomedical research. Microfluidic assays prove especially attractive as they allow precise control of gradient shape allied to a reduction in scale. Conventional microfluidic devices are fabricated using solid plastics that prevent direct access to responding cells. Fluid-walled microfluidics allows the manufacture of circuits on standard Petri dishes in seconds, coupled to simple operating methods; cell-culture medium sitting in a standard dish is confined to circuits by fluid walls made of an immiscible fluorocarbon. We develop and experimentally validate an analytical model of diffusion between two or more aqueous streams flowing at different rates into a fluid-walled conduit with the cross-section of a circular segment. Unlike solid walls, fluid walls morph during flows as pressures fall, with wall shape changing down the conduit. The model is validated experimentally for Fourier numbers < 0.1 using fluorescein diffusing between laminar streams. It enables a priori prediction of concentration gradients throughout a conduit, so allowing rapid circuit design as well as providing bio-scientists with an accurate way of predicting local concentrations of bioactive molecules around responsive and non-responsive cells.

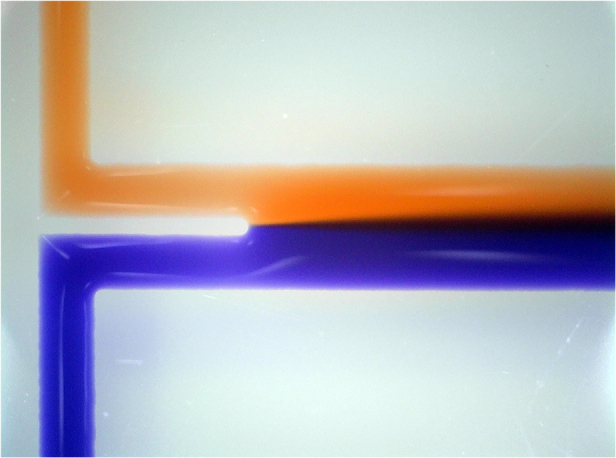

## Introduction

Diffusion represents fundamental mass transport, and many cellular responses are triggered by concentration gradients of specific molecules. For example, during a bacterial infection, macrophages circulating in the bloodstream exit vascular vessels to target the source of infection; such precise movement is driven by concentration gradients of secreted bacterial proteins^1^ and host hormones^2^. Similarly, migration of platelets towards wounds is driven by diffusion of subendothelial molecules into lacerated vessels where they activate the coagulation cascade^3,4^.

Despite the obvious importance of such phenomena, existing in vitro assays of cellular responses to molecular gradients have shortcomings. For example, chemotaxis is often studied using the transwell assay pioneered by Boyden^5,6^; however, diffusion gradients are unstable, the method is low throughput, and cells cannot be imaged as they respond in real-time. Recently, the introduction of microfluidic approaches has overcome many of these limitations^7^, but uptake of these methods remains poor^8^. Reasons cited for this include fabrication complexity^9^ and the inaccessibility of bio-samples contained behind solid plastic walls in devices that are often made of polydimethylsiloxane (PDMS). Consequently, more open microfluidic technologies are being developed^10,11^.

**Fig. 1:**
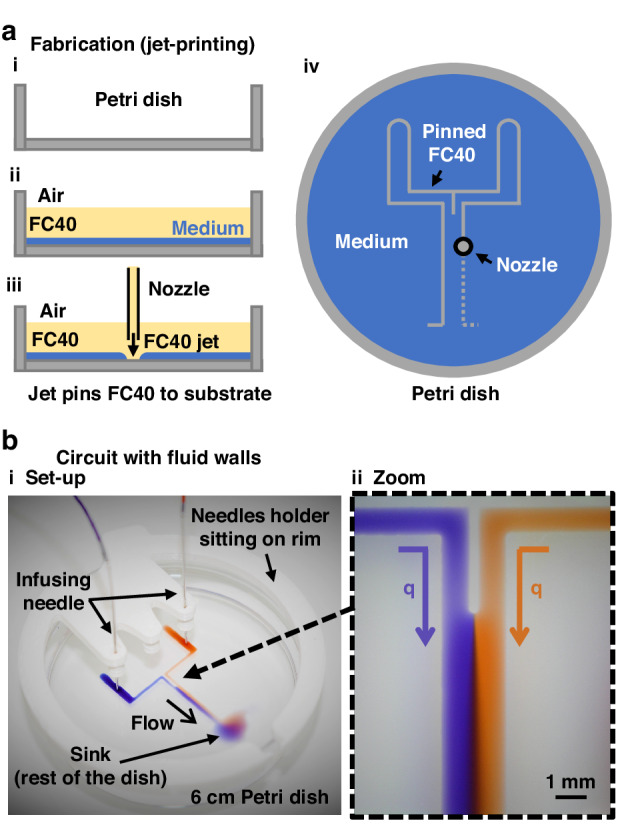
Making and operating a Y-shaped micro-circuit with fluid walls. **a** Fabrication by jet-printing. (**i**) A virgin polystyrene Petri dish. (**ii**) Add a thin layer of medium and quickly overlay with FC40. (**iii**) More FC40 is jetted through the overlay to locally sweep the medium away to leave FC40 pinned to the polystyrene substrate. (**iv**) A 3-axis traverse moves the jetting nozzle above the dish so it “prints” the Y-shaped footprint of the circuit without contacting polystyrene (dotted grey line: the future path of the jetting nozzle). **b** Operation. (**i**) Experimental setup. The circuit sits in a 6 cm dish, with a 3D-printed needle holder clipped onto the rim. After lowering two needles through the holder and into each arm of the Y, red and blue dyes are infused into the circuit (using an external syringe pump that is not visible); red and blue dyes merge at the junction in the Y to flow as laminar streams down the central arm, through the open end, and out into the sink (the rest of the dish). (**ii**) Zoom of central arm illustrating diffusion of blue dye rightwards, and red dye leftwards, between laminar streams (this gives a widening purple zone towards the bottom). *q* flow rate

In fluid-walled microfluidics^12^, the solid walls of conventional devices are replaced by liquid ones (i.e., interfaces between two immiscible phases). This counter-intuitive approach is possible due to specific properties of fluids at the micro-scale where gravitational effects become negligible, and interfacial forces govern interface geometry. This approach has been used to fabricate and operate microscale flow networks in simple cell-friendly ways^12–14^. For example, circuits are created in a standard Petri dish using a custom “fluid printer” that reshapes microscale volumes of the cell-culture medium under an immiscible and bio-inert fluorocarbon (FC40) (Fig. 1a). FC40 remains as an overlay on the immiscible fluid throughout the experiment to prevent evaporation. The printer consists of a 3D traverse equipped with a blunt needle (internal diameter $\sim 70\,{\rm{\mu }}{\rm{m}}$) connected to a syringe pump. A submerged jet of FC40 is pushed through the nozzle to sweep away the underlying medium in the dish to leave FC40 “pinned” to the substrate (Fig. 1aiii). The traverse moves the nozzle above the dish, without contacting media or dish, to reshape the aqueous phase into the desired pattern; such ‘jet-printing’ can make simple two-dimensional circuits in seconds^15–19^ (Fig. 1aiv).

The relevance of fluid-walled microfluidics to biology has recently been demonstrated by several biology groups^11–13,19–21^, with fluid-walled microfluidics successfully employed in chemotactic studies of bacteria^15^ and mouse macrophages^17^. In both cases, cells were exposed to stable diffusion gradients containing known chemo-attractants generated by flowing two parallel laminar streams. The fluid-walled circuits were used for several reasons, in preference to classical solid-walled devices, including the ability to isolate and retrieve cells that had undergone chemotaxis from the device prior to further downstream analysis like single-cell transcriptomics or proteomics^22–24^. While validated models exist for analogous circuits with solid walls like PDMS^25–27^, this paper provides an experimentally validated semi-analytical solution for devices with fluid walls.

To validate these models a ‘Y’-shaped circuit consisting of two inlet branches that converge into a single conduit and empties into a large sink (the rest of the dish) is used. At the junction, two inlet streams merge and flow side-by-side down the conduit as laminar streams, and the contents of these streams do not mix other than by diffusion across the inter-stream plane (Fig. 1bii). Superficially, this circuit resembles analogous ones made of PDMS^25–27^ (Fig, 1bi), but it differs in the important respect that the flexible and fluid walls morph according to pressure changes.

## Theory

### Morphing fluid walls

**Fig. 2:**
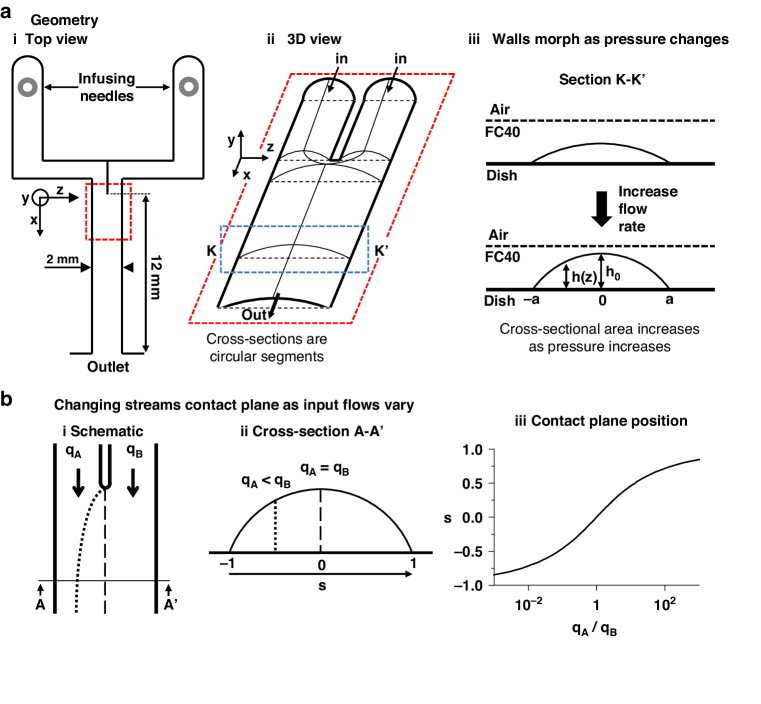
As flows change, conduit cross-sections change above unchanging footprints. **a** (**i**) Footprint of Y-shaped circuit. (**ii**) 3D schematic of the area within the dashed red line in (i); not to scale. As the circuit is bounded by fluid ceilings, cross sections of all aqueous arms are shaped like circular segments. (**iii**) Fluid walls morph as flows change to reflect pressure variations in an arm over an unchanging footprint, illustrated here at position K-K’—the area within the dashed blue line in (**ii**). The central height ${h}_{0}$ and cross-sectional area increase as the flow rate rises. **b** Changes in position of the contact line between laminar streams as input flow rates vary. (**i**) Top view of footprint at junction (${q}_{A}$ and ${q}_{B}$ are flow rates of the two inputs). Dashed and dotted lines: inter-stream contact planes when ${q}_{A}={q}_{B}$ (dashed), and ${q}_{A} < {q}_{B}$ (dotted). (**ii**) Cross-sectional view at position A-A’ in (**i**) showing two different locations ‘*s*’ of the contact plane at different input flow rates. (**iii**) Position ‘*s*’ of the contact plane across the width of the conduit for a range of inlet flow-rate ratios (log scale)

In the Y-shaped circuit, streams of two aqueous and miscible liquids converge at the junction to flow down the single straight conduit (width—2 mm, length—12 mm, height < 100 µm; Fig. 2ai). Conduit sections sit in the zy-plane, while the flow is along the *x*-axis. Our circuit sits in a standard polystyrene Petri dish and is capped by the interface formed with the overlaying fluorocarbon. Due to the fluid nature of the upper boundary, circuit cross-sections are shaped like segments of a circle (Fig. 2aii). During flow, the circuit footprint remains unchanged, but fluid walls/ceilings inevitably morph as pressures change. After fabrication, fluid walls and ceilings are initially flat above the circuit footprint, but when flow begins, they ‘inflate’ as a result of the increasing pressure. During steady flow, fluid walls stop morphing, but their height varies in the streamwise direction to reflect the local pressure in the conduit^18^. Consequently, the maximum height of conduit cross-section ${h}_{0}(x)$ decreases along the flow direction, being minimal at the outlet (Fig. 2aii). At different flow rates, ${h}_{0}(x)$ at any point down the conduit also differs (Fig. 2aiii).

Fluid dynamics at the microscale are typically characterised by low Reynolds numbers and laminar flows. This regime yields important simplifications and increased predictability of flows, allowing analytical solutions of the Navier-Stokes equations under well-defined boundary conditions. Recently, Deroy et al.^18^ derived a semi-analytical power law that describes the variation of ${h}_{0}$ down straight conduits of known geometry at constant flow rates (see Supplementary Information, and Supplementary Fig. 1), showing that flows at steady state can be modelled like those between infinite parallel plates. Then, using notations illustrated in Fig. 2aiii, one can derive equations of the flow velocity profile and of any cross-section profiles down the conduit:

1 $$\left\{h\left(z\right)=\sqrt{{\left(\frac{\left({a}^{2}+{h}_{0}^{2}\right)}{2{h}_{0}}\right)}^{2}-{z}^{2}}-\frac{{a}^{2}-{h}_{0}^{2}}{2{h}_{0}}\right.$$

2 $$\left\{u\left(y,z\right)=\frac{1}{2\mu }\frac{{{\rm {d}}P}}{{{\rm {d}}x}}\left({y}^{2}-\frac{{h\left(z\right)}^{2}}{4}\right)\right.$$

3 $$\left\{{u}_{\max }(z)=\frac{Q}{0.61{h}_{\left(z\right)}a}\right.$$

where $a$ represents conduit half-width, ${h}_{0}$ is the central height of the cross-section that varies along the *x*-axis as pressure decreases, $\frac{{{\rm {d}}P}}{{{\rm {d}}x}}$ and $u$ are the pressure gradient and local velocity of the fluid in the streamwise direction respectively, and ${u}_{\max }$ is the maximum velocity in a cross-section. ${u}_{\max }$ varies as $h$ changes along the *z*- and *x*-axes; it has a local maximum in each cross section at $z=0$: ${u}_{\max (0,x)}=\frac{Q}{0.61{h}_{0}a}$.

### Parallel laminar streams

When flow is laminar, the two input streams run side-by-side down the central conduit and mass transport between streams is by diffusion only. If the two inputs have identical viscosities, stream widths depend solely on the difference between the flow ratio of the input streams^28,29^ (Fig. 2b). Hence, the volumetric flow rate of each stream is given by the integral of velocity, $u(y,z)$, over the portion of the cross-section wetted by that fluid, and the ratio is

4 $$\begin{array}{c}\frac{{q}_{A}}{{q}_{B}}=\frac{{\int_{p}^{a}}{\int _{-\frac{h}{2}}^{\frac{h}{2}}}\frac{1}{2\mu }\,\frac{\partial P}{\partial x}\left({y}^{2}-\frac{{h\left(z\right)}^{2}}{4}\right){{\rm {d}}y{\rm {d}}z}}{{\int _{-a}^{p}}{\int _{-\frac{h}{2}}^{\frac{h}{2}}}\frac{1}{2\mu }\,\frac{\partial P}{\partial x}\left({y}^{2}-\frac{{h\left(z\right)}^{2}}{4}\right){\rm {d}}y{\rm {d}}z}\\ \therefore \frac{{q}_{A}}{{q}_{B}}=\frac{{\int _{p}^{a}}{h\left(z\right)}^{3}{{\rm {d}}z}}{{\int _{-a}^{p}}{h\left(z\right)}^{3}{{\rm {d}}z}}\end{array}$$

where $\pm a$ are the edges of the channel footprint, and ${q}_{A}$ plus ${q}_{B}$ are input flow rates, respectively (with total flow rate $Q={q}_{A}+{q}_{B}$). Every cross-section (normalised over the conduit half width) can be theoretically divided into two regions by a vertical line in position ‘$s=\frac{p}{a}$’ ($-1 < s < 1$) across its width representing the contact plane between streams (Fig. 2bii). However, given the variation of aqueous height across the conduit, there is no simple analytical equation predicting $s$—unlike the case for laminar flows in rectangular channels^28^. Therefore, a predictive numerical solution for ‘p’ is required; as expected, the trend is non-linear (Fig. 2biii).

### Diffusion gradients across parallel streams

Laminar flow and steady-state conditions significantly simplify modelling of mass transport between parallel streams, as diffusion is the only driving factor. The advection-diffusion equation at steady state for a solute (of concentration ${\boldsymbol{c}}$ and diffusion coefficient $D$) dissolved in an incompressible and isotropic fluid flowing with velocity ${\bf{v}}$ is

5 $$D{\nabla }^{2}{\boldsymbol{c}}-{\bf{v}}\cdot \nabla {\boldsymbol{c}}{\boldsymbol{=}}0$$

Considering unidirectional fully developed, laminar flow, along $x$, Eq. (5) simplifies Fick’s law as

6 $$\frac{\partial c}{\partial x}=\frac{D}{u}\frac{{\partial }^{2}c}{\partial {z}^{2}}$$

where $u$ is the velocity along the $x$-axis. Assuming diffusion between infinitely large reservoirs^30^, the solution to Eq. (6) is

7 $$c\left(z,x\right)=\frac{{C}_{0}}{2}{{\rm {erfc}}}\left(\frac{z}{2\sqrt{\frac{D}{\bar{u}}x}}\right)$$

We will use $\eta$ as the bracketed term. Diffusing molecules initially occupy a finite region, and the initial state is defined as $c={C}_{0}$ if $z\le 0$, and $c=0$ if $x \,>\, 0$. In Eq. (7), ${C}_{0}$ is the concentration of the solute in one of the inlet branches, and $\bar{u}$ represents the mean velocity of the parabolic profile (defined as $\bar{u}=\frac{2}{3}{u}_{\max }$). It is understandable from Eqs. (3) and (7) that concentration gradients do not simply depend on the position ($z,x$) but are affected by velocity changes along both $x$- and $z$-axes.

Finally, we define the flow time of molecules along the conduit $\left(t=\frac{L}{{\bar{u}}_{{{\rm {av}}}}}\right)$, where $L$ is the length of the conduit and ${\bar{u}}_{{{\rm {av}}}}$ the average of all mean velocities down the conduit of the contact plane between streams, and the diffusion time across the conduit $\left({t}_{{\rm {d}}}=\frac{{a}^{2}}{D}\right).$ The ratio of flow time over diffusion time defines the Fourier number (Fo):

8 $${{ {Fo}}}=\frac{t}{{t}_{{\rm {d}}}}=\frac{{DL}}{{a}^{2}{\bar{u}}_{{{\rm {av}}}}}$$

It represents a dimensionless contact time between streams flowing in the conduit; in other words, it defines the ratio of time molecules have to diffuse before reaching the outlet. We designed our circuit so that ${{ {Fo}}}\ll 1$ for all flow rates were tested. This condition allows observation of diffusion in the proximity of the contact plane between streams without altering inlet concentrations near conduit boundaries. In other words, as our model assumes that diffusion happens between infinite reservoirs, it is valid as long as ${{ {Fo}}}\ll 1$. Nevertheless, Eq. (8) represents the definition of ${{ {Fo}}}$ when input rates are equal, the contact plane sits in the middle ($s=0$), and the two streams occupy equal portions of the conduit. In cases where $s\ne 0$, we can define two Fourier numbers depending on which portion of the conduit is analysed. Thus, ${{ {Fo}}}=\frac{{DL}}{{[a(s+1)]}^{2}{\bar{u}}_{{\rm {m}}}}$ if ${q}_{A} \,>\, {q}_{B}$ and ${{\rm {Fo}}}=\frac{{DL}}{{[a(1-s)]}^{2}{\bar{u}}_{\rm {{m}}}}$ if ${q}_{A} \,<\, {q}_{B}$.

## Materials and methods

### Reagents

Cell culture medium used in this work is always Dulbecco’s modified eagle medium (DMEM, Sigma-Aldrich) supplemented with 10% foetal bovine serum (FBS, Gibco). The overlaying fluorocarbon (FC40) is purchased as 3 M Fluorinert^TM^ and subsequently treated (protocol property of iotaSciences Ltd) to obtain FC40STAR. The fluorescein solution used to calibrate measurements and observe diffusion gradients is prepared by dissolving fluorescein-dextran 9 kDa (FD-10S, Sigma-Aldrich) 300 µM in sterile PBS (phosphate-buffered saline, Gibco). Throughout this article, every time we mention medium/media, FC40, or fluorescein, we are referring to DMEM + 10%FBS, FC40STAR, and fluorescein-dextran 9 kDa in PBS respectively. The PBS-based solutions used have similar viscosities to any aqueous cell culture media, and so results should apply to all other culture media. Additionally, while PBS does not contain molecules that influence the fluorescence signal it is known that many media/sera do contain molecules known to increase or quench fluorescence signals.

### Microscopy and imaging

All experiments have been performed on an inverted microscope (Olympus IX53) equipped with a ×4 objective connected to a single-lens reflex camera (Nikon D7100 DSLR). Fluorescein molecules were excited by an LED light (CooLED *λ* = 470 nm, light intensity 15%) and fluorescent images were recorded with a shutter exposure of 0.25 s. All fluorescent images have been recorded after focusing on the FC40 walls pinned to the dish using phase contrast, then switching to fluorescence without adjusting the focus position. The image in Fig. 1bi was taken using a Nikon D5100 DSLR camera, and the one in Fig. 1bii with a Dino Capture 2.0 camera.

### Circuit fabrication

All circuits presented in this paper are jet-printed using standard clean polystyrene tissue culture-treated 60 mm Petri Dishes (Corning Inc, Life Sciences). First, the dish is filled with 1 ml cell culture medium to wet its surface, as much volume as possible is then carefully removed by pipet in order to leave just a thin layer wetting the surface. This aqueous layer is then immediately overlaid with ~5 ml of immiscible FC40, which forms a 2 mm layer that prevents evaporation. The dish is placed on a custom-designed fluid-shaping printer (iotaSciences). Then, the tip of a blunt needle (70 µm inner diameter; iotaSciences) held by the 3D traverse unit of the printer is lowered into the FC40 overlay until ~0.3 mm above the bottom of the dish, and additional FC40 jetted out of the needle at 480 µl/min (the needle is connected via a Teflon tube to a 1 ml glass syringe (Hamilton) driven by a syringe pump integrated into the printer). The jet sweeps the medium layer of the substrate to leave FC40 pinned to the dish. As the traverse moves the needle above the dish, the pinned FC40 forms “walls” that confine the aqueous phase in the desired circuit. The conduit is designed with a width of 2 mm, however as the pinned FC40 walls that bound the conduit have a thickness of ~150 μm, the actual conduit width is ~1.85 mm. Circuit patterns and printer control commands are written using G-code.

### Infusion pumps and tubing

All experiments are performed with syringe pumps (PhD ULTRA, Harvard Apparatus) equipped with two 100 µl glass syringes (Hamilton) connected to 25 G stainless steel blunt infusing needles (Hamilton) through 28 G Teflon tubes (Adtech). Needles are held vertically in position over inlet arms by home-made 3D-printed holders that clip on the rims of dishes (similarly to Deroy et al.^16^).

### Determining h(z) from fluorescence intensity

Fluorescence intensities in arbitrary units (a.u.) given by fluorescein seen in images are converted to local conduit height using linear calibration curves constructed as follows. A 2-inlet conduit is infused using the same fluorescein solution in both inlets at three different total flow rates (where ${q}_{A}={q}_{B}$), and nine images are recorded at every millimetre down the conduit between 2 and 10 mm from the junction. All images are then analysed using ImageJ (RRID:SCR_003070) to plot intensity profiles at each location across the conduit, and corresponding theoretical cross-section profiles are computed using Eq. (1) and divided by the pixel intensity in the same location ‘$z$’ to determine the height-to-intensity ratios. The three flow rates tested are 5, 10, and 20 µl/h (values refer to total flow rate $Q$).

### Determining diffusion gradients of fluorescein across conduits

In these experiments, PBS + fluorescein is infused into the left-hand arm and PBS into the right-hand one. Intensity profiles of flowing fluorescein are then measured across conduit width at every millimetre down the length of the conduit between 2 and 10 mm from the junction. Pixel intensity [a.u.] is converted into height [µm] using a linear calibration curve and subsequently divided by the theoretical height of the conduit cross-section (Eq. (1)) at the same location along the $x$-axis.

## Results

Mass transport by diffusion of fluorescein between parallel streams flowing through a straight fluid-walled conduit is observed by microscopy. The fluorescein solution is infused into one inlet branch (conventionally the left one), and PBS into the other one. After the junction, fluorescent molecules diffuse between laminar streams to yield increasing concentrations on the right as the distance from the junction increases. A fluorescence image of the conduit is collected, and intensity profiles of green fluorescence are then measured across the conduit width at every millimetre down its length between 2 and 10 mm from the junction. Then, the intensity profile is converted into an equivalent height profile, and local concentration is computed as the height ratio between the equivalent profile traced by fluorescein and the theoretical profile of the cross-section described by Eq. (1). In other words, if the height of the fluorescent equivalent profile at a specific location equals the theoretical one, no diffusion happened so the concentration there equals the infused concentration ${C}_{0}$.

### Deriving a calibration curve

Pixel intensity (a.u.) at a specified point in the resulting image must now be converted into a concentration, and this is usually achieved using a direct calibration done, for example, by measuring intensities of a dilution series of the fluorescein^31^. However, as bounding fluid walls/ceilings are not flat and morph as flow rates change, this induces the same concentration ${C}_{0}$ to correspond to multiple intensities depending on *z*-location and flow velocity. This prompted us to develop a calibration method that yields a linear curve applicable to all conditions used.

**Fig. 3:**
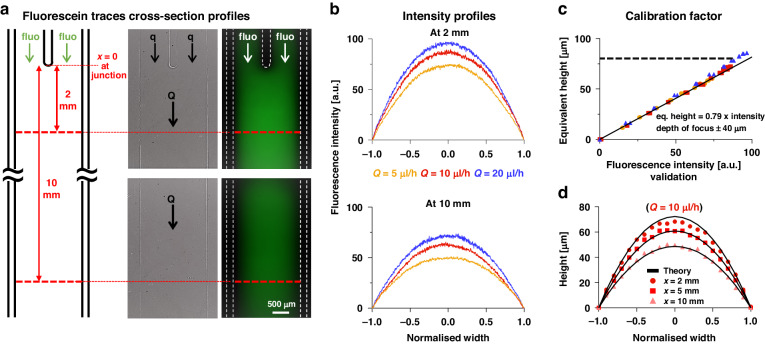
Calibration—a linear relationship between calculated height and fluorescent intensity. **a** Setup (Y-shaped circuit). After infusing fluorescein (fluo) into both input arms (${q}_{A}={q}_{B}$), images are collected along the conduit below the junction, and fluorescence intensities analysed across conduit widths every millimetre from 2 to 10 mm from the junction. Phase-contrast images with pinned FC40 walls visible. Fluorescence images show intensity decreases as pressures fall towards the outlet. **b** Fluorescence intensity (a.u.) profiles of conduit cross-sections at 2 and 10 mm from the junction for three different flow rates. **c** Linear relationship between fluorescence intensity and height. Data points are obtained by sampling intensity plots like those in (**b**) every 0.1 increments along the normalised width for all flow rates at the 2 mm cross-section, with colours reflecting flow rates in (**b**). (dashed black line represents the limit of the depth of field of the objective). **d** Plot of heights derived from intensities as in (**c**) against normalised width at 2, 5, and 10 mm from the junction. ($Q=10\,{\rm {{\mu l}/h}}$). Black curves: heights calculated from Eq. (1)

To develop the calibration method, the same fluorescein solutions are infused into both inlets so there is no gradient between streams (Fig. 3ai), and images of the conduit are collected with the focus on the base of the fluid walls pinned to the dish. These pinned walls are visible in phase-contrast images (Fig. 3aii), but not in fluorescence ones (Fig. 3aiii), where they are shown as dashed white lines here and in subsequent images. Two trends are visible in fluorescence images: intensity increases between 0 and 2 mm from the junction ($x=0$ at the junction), before progressively declining towards the exit. The increase is due to the sudden change of width and flow rate that happens at the junction. Fluid walls/ceiling height lift to accommodate such changes (~4-fold increment), hence a brighter intensity is visible. Such height variation is not immediate but occurs over the first couple of millimetres after the junction; however, the complexity of the curvature of the fluid walls/ceilings in this section does not allow analytical prediction (Supplementary Fig. 1). Consequently, Eq. (1) do not apply within 1-2 mm of the junction^18^, and we sample intensities every millimetre from 2 to 10 mm (Fig. 3b shows intensity profiles at 2 and 10 mm from the junction for three different flow rates). As conduit heights vary to balance pressure, intensity profiles decrease towards the outlet; they also increase as the flow rate increases. Next, intensities are sampled in 0.1 increments across the normalised width of the cross-section (for all three flow rates) and plotted against conduit heights calculated from Eq. (1). They fall on a straight line with slope $0.79\pm 0.03$. This line is derived from the use of intensities in all pixels measured ($n=\mathrm{45,495}$) across the nine cross sections and for the three flow rates (Fig. 3c). In other words, there is a linear relationship between height [µm] and pixel intensity [a.u.]—where equivalent height = 0.79 × intensity—over a wide range of conditions. Theory fits well with experimental points inside the depth of field of the objective (~80 µm, $\pm 40{\rm{\mu }}{\rm{m}}$ around the focal plane; manufacturer’s data) and is expected to diverge outside this range. Therefore, this linear approximation enables prediction of all heights across the width of the conduit at different distances from the junction (Fig. 3d), although errors progressively increase when height exceeds the depth of field (Fig. 3d, compare red circles with upper black line) and this becomes a limiting factor of the method.

### Diffusion gradients across parallel streams

**Fig. 4:**
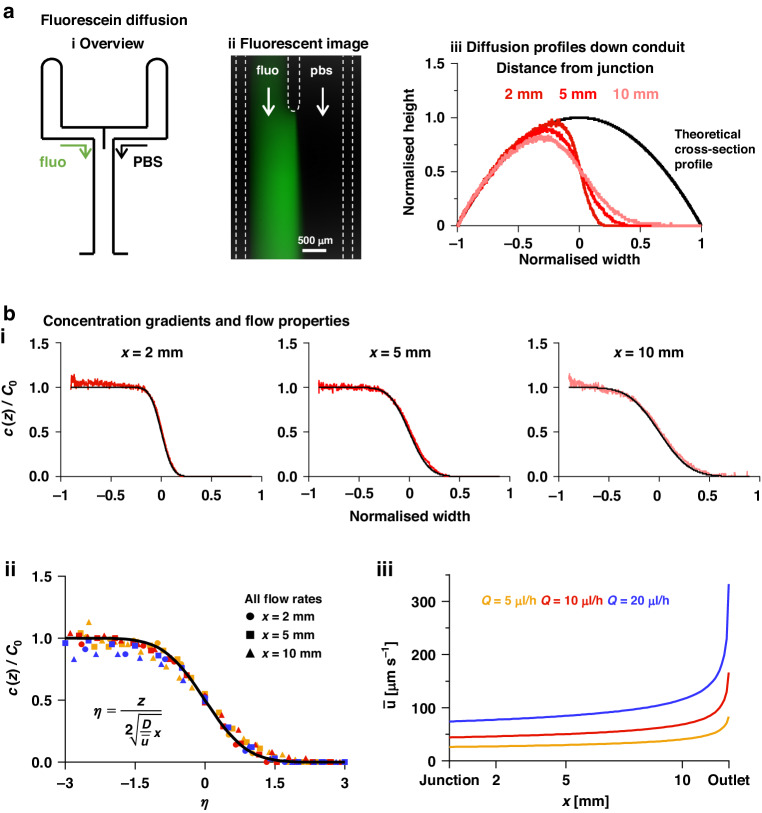
Diffusion of fluorescein from one laminar stream into a fluorescein-free stream flowing at the same rate. **a** Overview. (**i**) Schematic. PBS-fluorescein (fluo) is inputted into the left-hand arm, and PBS into the right-hand one (*Q* = 10 µl/h; ${{\rm{q}}}_{{\rm{A}}}={{\rm{q}}}_{{\rm{B}}}$). (**ii**) Representative fluorescent image of the junction. Dashed lines mark fluid walls pinned to the dish. (**iii**) Diffusion profiles across the central conduit at positions 2, 5, and 10 mm from the junction. For each distance from the junction, there is one black curve (derived using Eq. 1) plus an associated red curve (derived from intensities measured in images like that in (A)ii, and then converted to equivalent heights using the calibration factor). Pairs of curves are overlaid by normalising widths and heights with respect to ${h}_{0}$ on each black curve. **b** Concentration gradients and flow properties. (**i**) Concentration profiles (red) measured 2, 5, and 10 mm from the junction compared to predictions from Eq. (7) (black line). Experimental gradients are computed as the ratio of a diffusion profile (shown in **Aiii**) and the corresponding cross-section profile at each point across the width. (**ii**) Collapsed experimental data for all flow rates tested (for $\eta$ see Eq. (7); colours refer to flow rates and shapes of data points indicate distances from the junction) and predicted curve from Eq. (7) (black line). (**iii**) Mean velocities in the centre of the conduit for different flow rates (from Eq. (3))

We now return to the original setup where fluorescein in PBS, and just PBS, are infused into the left- and right-hand input arms to flow as laminar streams down the central arm (Fig. 4ai). Soon after the junction, fluorescein diffuses across the contact plane between the two laminar streams (Fig. 4aii). We quantify diffusion by recording pixel intensity across the conduit at *x* = 2–10 mm as before, and convert intensities to equivalent heights using the calibration factor (Fig. 3c). The equivalent height profile across the conduit can be paired with a related one derived from Eq. (1). Such pairs are now overlaid by normalising widths and heights with respect to $s=0$ and ${h}_{0}$ (Fig. 4aiii). Equivalent-height profiles perfectly follow the theoretical ones on the left of the conduit, and—in the absence of diffusion—they should fall immediately to zero (at normalised width 0) in accordance with Eq. (7); instead, they decline gradually. Each of the resulting profiles is equivalent to the corresponding concentration profile, as the ratio of equivalent to theoretical heights (red to black in Fig. 4aiii) at each point across the conduit reflects the local fluorescein concentration. Thus, where the equivalent height equals the theoretical one, the fluorescein is undiluted (100% ${C}_{0}$); where the ratio is zero, there is no fluorescein (0% ${C}_{0}$). When concentrations derived from intensities in this way are compared to the predictive model (Eq. (7)), there is excellent agreement across conduits at all three flow rates (Fig. 4bi, Supplementary Fig. 2). All results obtained from theory and experiment are now collapsed into one chart (Fig. 4bii); the excellent convergence between the two validates the theory for predicting diffusion profiles as velocities vary down a conduit (Fig. 4biii).

**Fig. 5:**
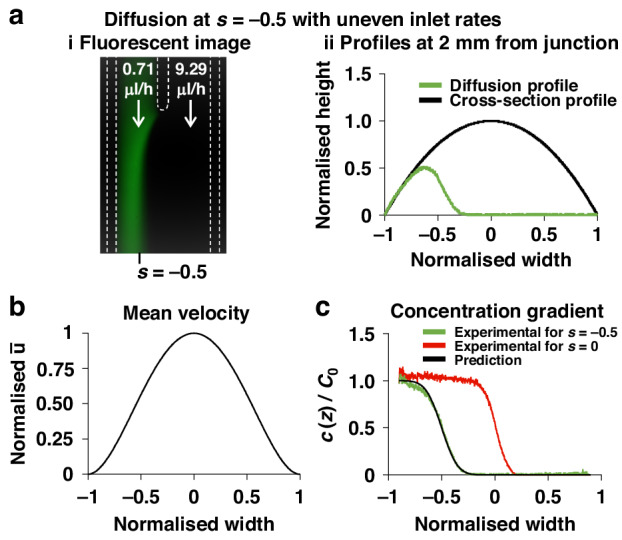
Diffusion of fluorescein between laminar streams with different flow rates. **a** Diffusion profile for uneven streams with contact line shifted to $s=-0.5$. Conditions to achieve this shift—with $\frac{{q}_{A}}{{q}_{B}} \sim {10}^{-1}$ and $Q={q}_{A}+{q}_{B}=10{\rm{\mu }}{\rm{l}}/{\rm{h}}$—are determined using Fig. 2**b**iii. (**i**) Representative fluorescent image. (**ii**) Experimental diffusion profile (converted from intensity) 2 mm from the junction compared to the theoretical one (from Eq. (1)). **b** Normalised plot of mean velocity 2 mm from the junction. Unlike rectangular conduits, the velocity profile across the width of the conduit is not parabolic. **c** Diffusion/concentration profiles (green) measured 2 mm from the junction—derived as in Fig. 4**b**i—compared to predictions from Eq. (7) (black line). The red curve is reproduced from Fig. 4**b**i

The position of the contact plane between parallel streams can also be controlled precisely in our system using Eq. (4). We illustrate this by moving the contact plane away from the centre of the conduit to position $s=-0.5$. Thus, setting $\frac{{q}_{A}}{{q}_{B}}=0.0763$ (and $Q={q}_{A}+{q}_{B}=10{\rm{\mu }}{\rm{l}}/{\rm{h}}$) should induce the required shift (Fig. 2biii)—and it does (Fig. 5ai). Conversely, in solid-walled conduits with fixed height, the same movement would be achieved with $\frac{{q}_{A}}{{q}_{B}}=0.25$ (Fig. 5b). Therefore, corrections for local velocities, $\bar{u}\left(s,x\right)$, are included when computing concentration profiles using Eq. (7); again, there is good correspondence between theory and experiment.

### Diffusion gradients across three parallel streams

**Fig. 6:**
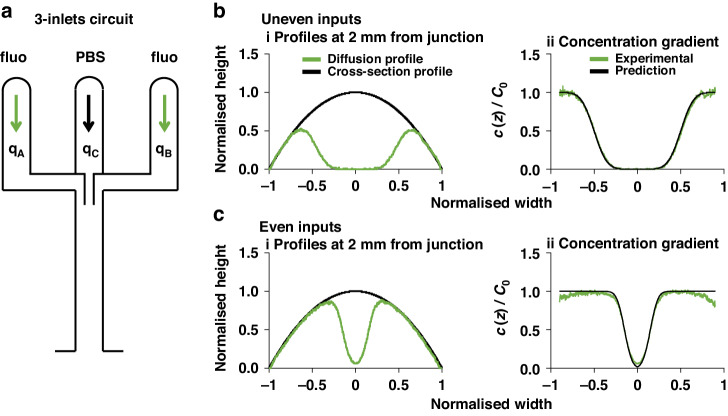
Diffusion of fluorescein between three laminar streams with varying flow rates. **a** Schematic of the circuit—a trident with three inlets. Fluorescein and PBS alone are inputted as shown. **b** Diffusion gradients with uneven inputs $({q}_{A}={q}_{B}=0.71\mu l/h,{q}_{C}=8.58\mu l/h,Q=10\mu l/h)$. Contact planes between streams fall at ${s}_{1}=-0.5,$ and ${s}_{2}=0.5$ $\left({{\rm {using}}}\frac{{q}_{A}}{{q}_{C}+{q}_{B}} \sim {10}^{-1},\frac{{q}_{A}+{q}_{C}}{{q}_{B}} \sim {10}^{1},{{\rm {and}}\; Q}={q}_{A}+{q}_{C}+{q}_{B}=10\mu l/h\right)$. (**i**) Diffusion profile 2 mm from the junction. (**ii**) Concentration profiles (green) measured 2 mm from the junction (derived as in Fig. 4**b**i) compared to predictions from Eq. (7) (black line; the solution across the contact plane in the $0 < {s}_{2} < 1$ range is that for ${s}_{1}$ mirrored around $s=0$). **c** Diffusion gradients with even inputs. $({q}_{A}={q}_{C}={q}_{B}=6.67\mu l/h,Q=20\mu l/h)$. Contact planes between streams fall at ${s}_{1}=-0.15$, and ${s}_{2}=0.15$ $\left({{\rm {using}}}\frac{{q}_{A}}{{q}_{C}+{q}_{B}}=0.5,\frac{{q}_{A}+{q}_{C}}{{q}_{B}}=2,{{\rm {and}}}\,{q}_{A}+{q}_{C}+{q}_{B}=20\mu l/h\right)$. (**i**) Diffusion profile 2 mm from the junction. (**ii**) Concentration profiles (green) measured 2 mm from the junction (derived as in Fig. 4**a**i) compared to predictions from Eq. (7) (black line, mirrored as above)

Finally, results from theory and experiment are compared using an extra inlet to give three laminar streams in the central conduit—with two fluorescein streams flanking a central PBS one (Fig. 6a). We position contact plane ${s}_{1}=-0.5$ (as Fig. 5), and ${s}_{2}=0.5$, by setting $\frac{{q}_{A}}{{q}_{C}+{q}_{B}}=0.0763$, $\frac{{q}_{A}+{q}_{C}}{{q}_{B}}=13.09$, and $Q={q}_{A}+{q}_{C}+{q}_{B}=10{\rm{\mu }}{\rm{l}}/{\rm{h}}$. The normalised equivalent-height profile now has a (green) peak at each edge (compare Fig. 6bi with Fig. 5aii), and the (green) concentration-gradient profile is both symmetrically placed around $z=0$ and overlaps the predicted one (Fig. 6bii). We also equalise flow rates using $\frac{{q}_{A}}{{q}_{C}+{q}_{B}}=0.5$, $\frac{{q}_{A}+{q}_{C}}{{q}_{B}}=2$, and $Q={q}_{A}+{q}_{C}+{q}_{B}=20{\rm{\mu }}{\rm{l}}/{\rm{h}}$ (Fig. 6c). This sets ${s}_{1}=-0.15$ and ${s}_{2}=0.15$ and so should narrow the central stream; it does (Fig. 6ci), and there is again symmetry plus good correspondence between theory and experiment (Fig. 6cii).

## Discussion

The fluid nature of walls in our micro-circuits (Fig. 1) ensures that conduit cross-sections are circular segments (Fig. 2aii) that morph above unchanging footprints with changing pressure (Fig. 2aiii). This is unlike the unchanging cross-sections found in most conventional circuits with solid walls. Deroy et al.^18^ showed such behaviours, proving the cross-sectional area reduces from inlet to outlet due to pressure gradient (Supplementary Fig. 1). They derived a power-law equation that predicts heights of conduit in the flow direction under constant flow rate when confined by fluid walls (Eq. (S1)).

We begin with a Y-shaped circuit, infuse inputs into the two lateral arms, and monitor laminar flows in the central conduit (Fig. 2ai). In each straight section, the model proposed by Deroy et al. can be applied, so flow and fluid wall dynamics are fully described by Eqs. (1)–(3). When a solute (fluorescein-dextran of 9 kDa) dissolved in PBS is steadily infused into the left-hand arm and PBS into the right-hand one, after convergence the solute diffuses between the laminar streams. As the total flow rate is the sum of all inputs ($Q=\sum {q}_{{{\rm {inlet}}}}$), input ratio defines the stable contact plane between streams (Eq. (4)).

As in previous studies^17,26,27^, solute transfer between streams is now modelled assuming one-dimensional diffusion between infinitely large reservoirs (Eq. (7)). However since cross-sectional areas down the conduit vary, flow velocities on the contact plane also do so; this is a unique characteristic of our system. Moreover, before flow begins, all parts of a circuit share the same negligible internal pressure and fluid walls lie relatively flat over the footprint. However, once flows begin, pressures increase, and walls morph to reach the shape described by Eq. (1). The time required for flow to reach steady state (${t}_{{{\rm {start}-{up}}}}$), mostly depends on the geometry of the circuit and on inlet flow rates. Steady state is achieved when fluid walls stop morphing and ${Q}_{{{\rm {in}}}}={Q}_{{{\rm {out}}}}$. The minimum time required to reach a steady state may be approximated as the time necessary for an equivalent volume to the one contained in the circuit found at the steady state to flow through; ${t}_{{start}-{up}}=\frac{{V}_{{circui}{t}_{{steady}-{state}}}}{Q}$ (i.e., minimum time to fill the circuit assuming ${Q}_{{out}}=0$). In our circuit geometry and with the slowest flow rate tested (${q}_{A}={q}_{B}=2.5{\mu}{\rm{l}}/{\rm{h}},{so Q}=5{\mu}{\rm{l}}/{\rm{h}}$), ${t}_{{start}-{up}(\min )}=93$ min which is in reasonable agreement with experimental results shown in Supplementary Fig. 3, which showed steady-state conditions are reached after ~2 hours (a range of circuit geometries and startup times are shown in Supplementary Fig. 4). Therefore, all measurements are made at least 3 h after flow begins to establish the steady state that the persists subsequently for at least 10 h in our experiments providing flow rates remain unchanged. (Supplementary Fig. 3).

Diffusion of our solute between laminar streams is monitored by fluorescence microscopy, and intensities seen in images are converted to concentrations using a linear calibration curve that applies to all conditions used – provided that conduit heights lie within the depth of field of our microscope (Fig. 3 and Supplementary Fig. 5). After inputting equal flows into each inlet (${q}_{A}={q}_{B}$), and after varying total flows into the circuit ($Q=\mathrm{5,10},{\rm{or}}\,20\mu {\rm{l}}/{\rm{h}})$, diffusion profiles determined experimentally match those predicted using Eq. (7) down the length of the conduit (Fig. 4). Use of a diffusion coefficient for fluorescein-dextran 9 kDa (${D}_{\exp }=1.1\times {10}^{-10}{{\rm{m}}}^{2}/{\rm{s}}$) provides the best fit with experimental data (Supplementary Fig. 2); this is in reasonable agreement with the theoretical value computed with the Stokes–Einstein equation (${D}_{{{\rm {th}}}}=\frac{{k}_{{\rm {B}}}T}{6\pi \mu R}=1.07\times {10}^{-10}{{\rm{m}}}^{2}/{\rm{s}}$), where ${k}_{\rm {{B}}}$ is the Boltzmann constant, $T$ is room temperature (298.15 K), $\mu$ is the dynamic viscosity of the solution assumed to be that of water (0.89 cP), and $R$ is the radius of the diffusing molecule (23 Å)^32^.

Theory is also validated in two additional ways. In one, inputs are infused into the two arms of the Y-shaped circuit at different rates—ones that are predicted to shift the contact plane between laminar streams away from the centre line to a new specified position. Although such a shift changes the mean velocity profile, experiment showed it occurs as expected to yield the appropriate concentration gradient (Fig. 5). The second way involved a trident-shaped circuit with three inlet arms (Fig. 6a). After inputting fluorescein into flanking inlets and PBS into the middle one, three laminar streams now flow side-by-side to yield two contact planes; again, predicted and experimentally-determined gradients overlap (Fig. 6b, c).

Finally, our model assumes diffusion across contact planes occurs between infinitely large reservoirs; in other words, we assume flow is significantly faster down the conduit compared to lateral diffusion so that diffused molecules do not affect bulk concentrations in a neighbouring stream. The Fourier (${{\rm {Fo}}}$) number (Eq. (8)) is the ratio of diffusion and flow times. We use ${\rm {{Fo}}} < 0.1$. Thus, for all experiments with two inlet streams and even input flow rates (Fig. 4, Supplementary Fig. 2), ${{\rm {Fo}}}$ ${\rm{is}} \sim {10}^{-2}$ for all flow rates tested; then, the model correctly predicts diffusion gradients. However, once the contact plane shifts away from the centre line, our model fits experimental data only at *x* = 2 mm, and becomes progressively less accurate at greater distances from the junction (Fig. 5). For example (Supplementary Fig. 6a), where the fluorescent stream is narrow and the contact plane is close to the left edge of the conduit ($s=-0.5$), and its velocity falls (Fig. 5b) to become comparable to the velocity of diffusing molecules; therefore, the initial bulk concentration of fluorescein falls below ${C}_{0}$ and Eq. (7) no longer holds. In this configuration, when $Q=10{\rm{\mu }}{\rm{l}}/{\rm{h}}$, ${{\rm {Fo}}} \sim 0.25$ and theoretical results diverge from experimental ones. Similarly, when $Q=20{\rm{\mu }}{\rm{l}}/{\rm{h}}$, ${{\rm {Fo}}} \sim 0.15$ and our model accurately predicts the gradient up to 5 mm from the junction, but not further away (Supplementary Fig. 6a). Moreover, when contact planes in the trident are close to the centre of the conduit and the central stream of PBS is narrow (Supplementary Fig. 6b), the predictive model again performs poorly as solute from both sides alters the concentration in the central PBS.

In conclusion, we suggest the experimentally validated theory in this work represents a useful tool to design innovative fluid-walled microfluidic platforms for in-vitro studies on cell chemotaxis^15,17^, where one of the many advantages is the ability to reconfigure fluid walls and thereby isolate migrating cells^16^. Such studies require knowledge of local concentration along gradients and their steepness, and our model precisely describes diffusion between parallel streams flowing through conduits bounded by fluid walls. This work initially provides an equation to quantify the portion of the conduit occupied by each stream as a function of their flow-rate ratio [Eq. (4)]. Then, it establishes a model that accurately quantifies the concentration gradients of molecules of known diffusivity between such streams even when velocity profiles and conduit heights vary [Eq. (7)]. The ability to predict these gradients should facilitate the rapid development of new or more complex assays (Supplementary Fig. 7) and—when combined with the ability to easily retrieve any cells that have migrated from the circuit—provide a unique experimental platform for chemotactic studies.

### Supplementary information

Supplementary Information
