# Supplementary material for: Stable diffusion gradients in microfluidic conduits bounded by fluid walls
Source: Microsyst Nanoeng. 2024 Jun 20;10:79. doi: 10.1038/s41378-024-00698-1 (PMC11189932; doi:10.1038/s41378-024-00698-1)
Supplement: Supplementary file 1 — Supplementary Information [file 41378_2024_698_MOESM1_ESM.docx]

**Supplementary Information**

**Semi-analytical solution for** $\boldsymbol{h}_{\boldsymbol{0}}\left( \boldsymbol{x} \right)$

As liquid interfaces are flexible and can expand or retract, the fluid walls/ceilings in our circuits can inflate or deflate over the fixed footprint as inner pressures change. Variation in the shape of the walls changes conduit cross-section, and modifies flow velocities. Recently, Deroy et al.^18^ derived a power law that describes changes of the central height ($h_{0}$) of a straight fluid-walled conduit along its length ($L$) when a fluid with constant viscosity ($\mu$) flows at constant rate ($Q$) through it:

|  | $h_{0}\left( x \right)=\left( \frac{26.08\mu aQ\left( L-x \right)}{\gamma}+h_{out}^{4} \right)^{0.25}$ | (S1) |
| --- | --- | --- |

Here, $a$ represents the half width of the conduit, $\gamma$ the interfacial tension at the medium-FC40 interface, while $h_{out}$ is the central height of the conduit outlet. These authors also proved this equation to be valid only for conduits with high aspect ratios ($a\gg h_{0}$).

We build on this work and use Eq. S1 to describe $h_{0}$ along conduit length for all flow rates tested, and in Eq. (1), (2), and (3). From calibration intensity profiles that outline shapes of conduit cross sections, we derive the central intensity (at the highest point of the cross section) as the average of all intensity in the centre of the section ($-0.1<z<0.1$). We convert intensity into equivalent heights $h_{0}\left( x \right)$ using the linear relationship described in Figure 3, and find good agreement between experiment and theory for heights less than the depth of field of the microscope (Supplementary Fig. 1).

**Maximum flow rate** $\boldsymbol{(Q}_{\boldsymbol{max}}\boldsymbol{)}$

Deroy et al.^18^ derived Eq. S1 modelling the flow as between infinite parallel plates, and they showed this assumption holds as long as $\frac{h_{0}}{a}\leq0.2$. As a consequence, the diffusion model presented here is valid if the above condition is respected. In our circuit, $h_{0}$ is maximal just after the junction $(x \sim2 mm;$ Supplementary Figure 1B); therefore, we can derive a simple equation that describes the maximum flow rate allowed:

|  | $h_{0}^{4}\left( x=2 \right)=\frac{26.08\mu aQ(L-2)}{\gamma}, h_{0}\left( x=2 \right)=0.2a$  $\therefore Q_{max}=\left( 6.135 \times{10}^{-5} \right)\frac{\gamma a^{3}}{\mu(L-2)}$ | (S2) |
| --- | --- | --- |

For the circuit used in the paper with $a=0.925 mm$, $L=12 mm$, $\gamma=23 mN\cdot m^{-1}$, and $\mu=0.89 cP$ (assuming the viscosity of PBS as that of water at room temperature), it results $Q_{max}=450 \mu l\cdot hr^{-1}$. Despite the model presented being correct as long as $Q<Q_{max}$, experimental results are limited by the depth of field (DoF) of the objective used. Therefore, conditions were selected so as to guarantee the maximum measured height of the conduit, $h_{0}\left( x=2 \right)$ never exceeds the DoF. The following table summarises conduit heights at $x=2$ mm from the junction for the different flow rates tested. One can appreciate that for $Q=20 \mu l\cdot hr^{-1}$ the maximum height of the conduit matches the DoF, while faster flow rates would introduce measurement distortions.

| **Q [**$\boldsymbol{\mu l/hr]}$ | $\boldsymbol{h}_{\boldsymbol{0}}\boldsymbol{(x=2)}$ | **depth of field** |
| --- | --- | --- |
| 5 | 60 $\mu m$ | ~ 80 $\mu m$ |
| 10 | 71 $\mu m$ |  |
| 20 | 84 $\mu m$ |  |
| 450 | 184 $\mu m$ |  |


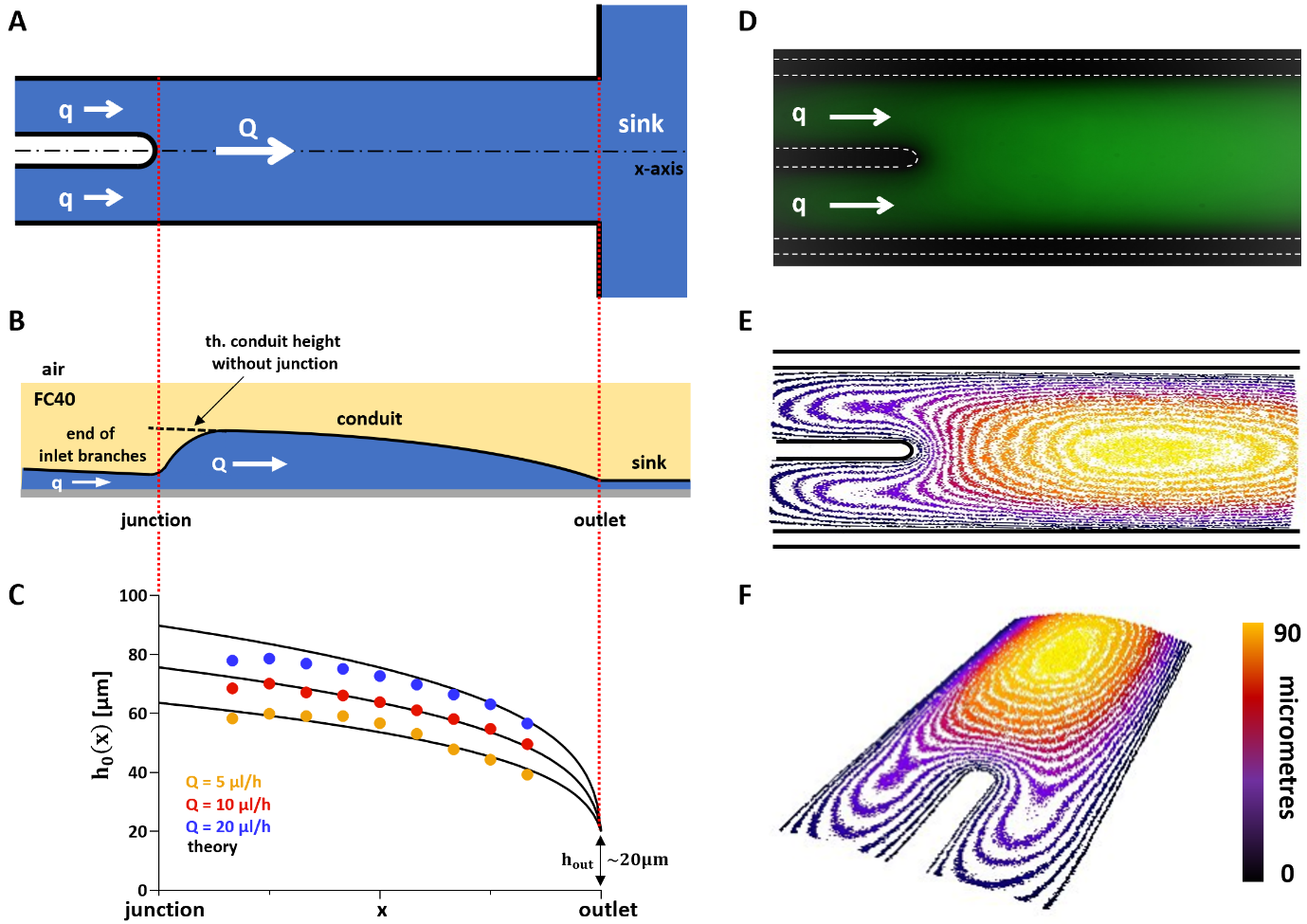


**Supplementary Figure 1. Central height profile of the conduit -** $\boldsymbol{h}_{\boldsymbol{0}}\boldsymbol{(x)}$**.**

**(A)** Top-view schematic of the circuit.

**(B)** Side-view schematic of the circuit. At the junction, a sudden change in geometry (inlet branches merge into single conduit with doubled width) induces fluid walls/ceiling to raise. Such variation is not immediate but occurs over 1-2 mm. At around 2 mm from the junction fluid walls/ceiling reach the height predicted by Eq. (S1) and shape described by Eq. (1)

**(C)** Experimental data compared to the analytical prediction of the central height profile of the conduit. Experimental data (circles) are values of $h_{0}(x)$ determined from central intensities of cross-section profiles between 2 and 10 mm from the junction for the flow rates indicated, while black lines are the respective theoretical predictions calculated using Eq. (S1).

**(D)** Representative fluorescent image with both input rates of 10 $\mu l/h$ (same as in Fig. 3A).

**(E)** Intensity isoline chart of fluorescent image in (D) analysed with ImageJ. It shows fluorescent intensity to gradually increase after the junction reflecting variations in fluid walls/ceilings.

**(F)** 3D isoline chart (ImageJ) with intensity converted into equivalent heights.


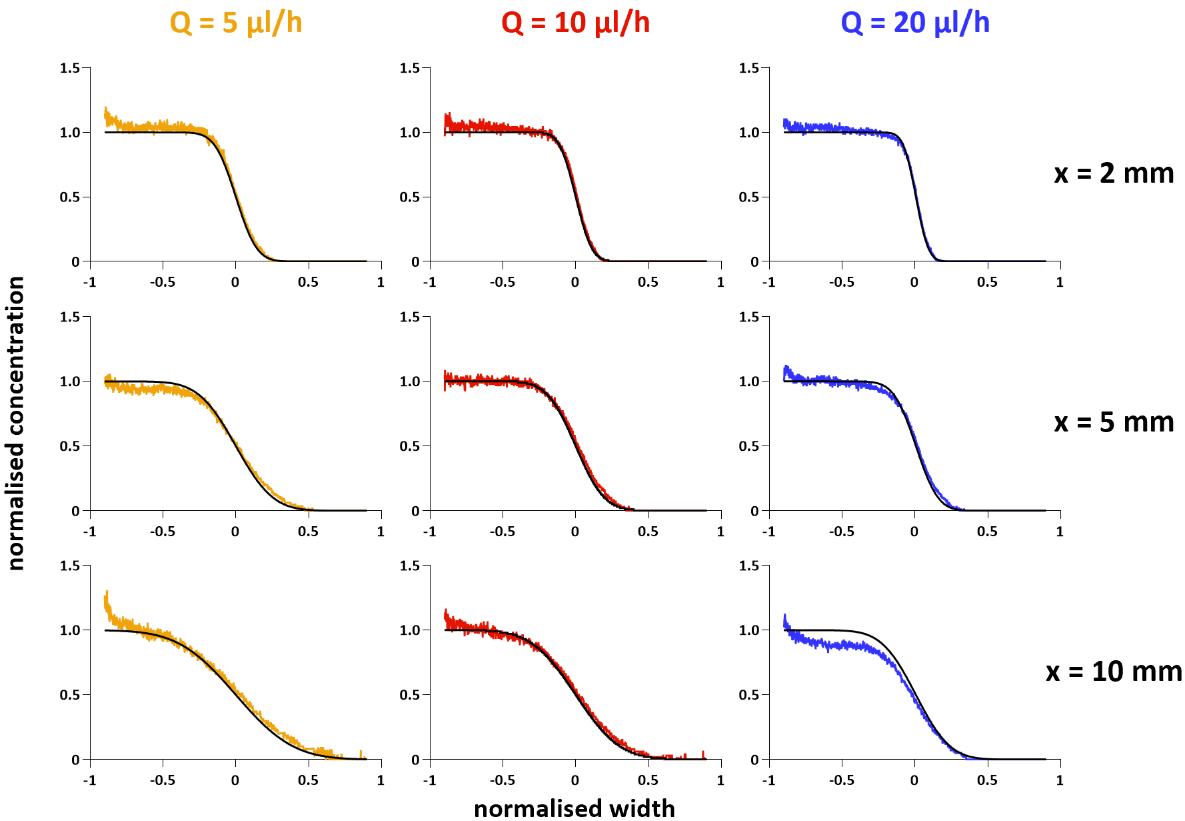


**Supplementary Figure 2. Diffusion gradients down the central conduit for all flow rates tested (Y-shaped circuit, conditions as Fig. 4).**

As input rates are equal, the contact plane between streams is centred in the conduit and diffusion is symmetric across its width. As expected, gradient steepness decreases towards the outlet ($x_{\mathrm{out}}=12 mm$), but increases at higher rates. Values for $Q=10 \mu l/h$ are reproduced from Figure 4B.


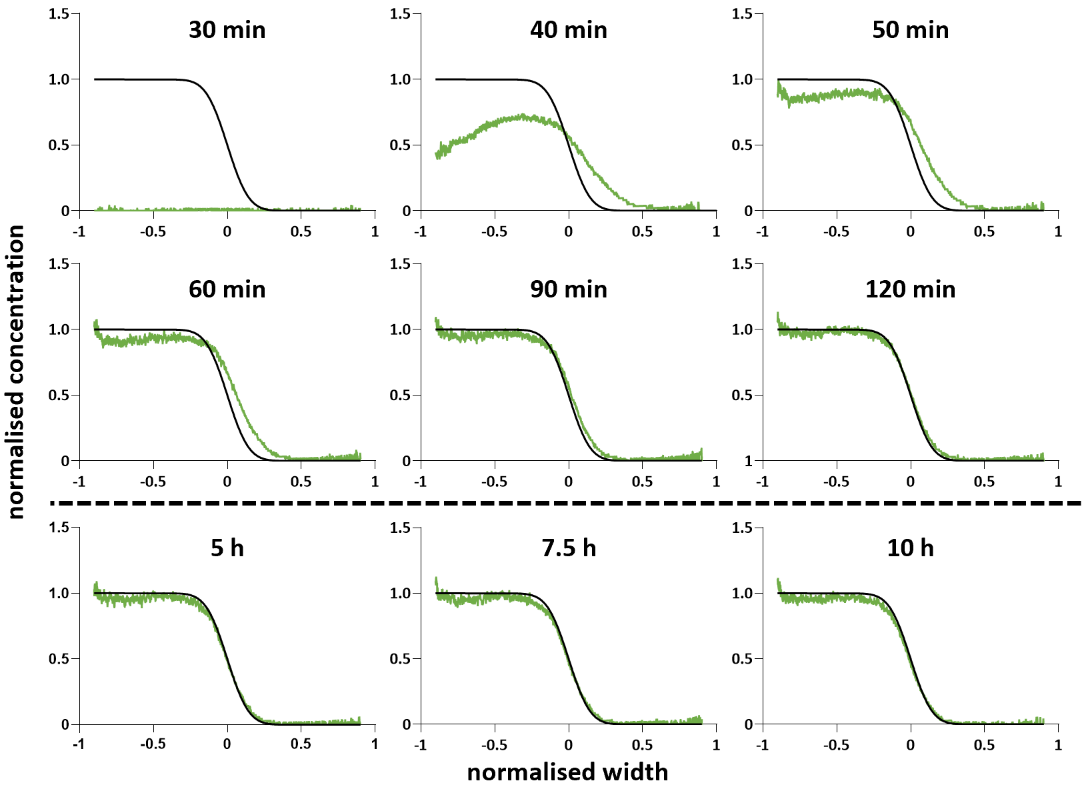


**Supplementary Figure 3. Start-up time and stability of diffusion gradients.** Conditions are as described in Figure 4B except that Q = 5 µl/h with $q_{A}=q_{B}$). Graphs show a time-lapse sequence describing the evolution of the concentration gradient across conduit cross-section at 2 mm from the junction. Infusing needles are inserted in the respective input arms and flow started. Time is counted from the moment both samples start releasing from infusing needles, measurements are recorded every 10 min, and concentration gradients determined (as in Fig. 4B). After 40 minutes, infusing samples reach the junction and start forming the diffusion gradient across streams. After 2 hours, the gradient reaches its final configuration, in perfect agreement with theoretical predictions (indicated by black lines). After this time, the diffusion gradient remains stable and unchanged. Flow is then stopped after 10 hours. We note that the time taken to reach steady state depends mainly on the geometry of inlet branches and input flow rates, however these dependencies were not explored further in this study.

**Estimation of time required to reach steady state**

The precise quantification of the time in which flow reaches steady state is a complex analysis that would require derivation of time-dependent equations. Nevertheless, it represents an important parameter in mass-transport phenomena as it determines the time required for diffusion gradients to reach stability. In this paragraph, we present a simple solution to allow users to quickly estimate the start-up time of flows through fluid-walled circuits like the one presented.

At steady state, fluid walls reach their stable configuration described by Eq. S1. This entails the circuit has an unchanging 3D shape and therefore “contains” an unchanging volume. We defined the start-up time as the time necessary to flow through the circuit an equivalent volume as the one occurring contained in the circuit at steady state. This translates as:

|  | $t_{start-up}=\frac{V_{circuit}}{Q}$ | (S3) |
| --- | --- | --- |

We subdivided the length of our circuit into three sections (Supplementary figure 4A): (i) the conduit (straight section after the junction, green, $a_{conduit}=1 mm$ and $L_{conduit}=12 mm$), (ii) the inlet (straight section that hosts the infusing needle, blue, $a_{inlet}=1 mm$ and $L_{inlet}=12 mm$), and the branch which connects inlets to the conduit (yellow, $a_{branch}=0.5 mm$ and $L_{branch}=12 mm$). We then calculated the central height of fluid walls in each section ($h_{0}$) using Eq. S1. The same equation does not apply to transition areas where width changes (red lines in Supplementary figure 4A), however Deroy et al.^18^ proved that the final height of each section is proportional to the quadratic ratio of the widths. Plot in Supplementary figure 4B shows the profile of the central height of fluid walls when $Q=2\cdot q=5 \mu l h^{-1}$. Knowing $h_{0}(x)$ and $a$ of each section, one can compute cross-sectional areas as:

|  | $A\left( x \right)=\frac{\left( a^{2}+h_{0}^{2}(x) \right)^{2}}{4h_{0}^{2}(x)}\cdot arcsin \left( \frac{2ah_{0}\left( x \right)}{a^{2}+h_{0}^{2}\left( x \right)} \right)-a\cdot\frac{a^{2}-h_{0}^{2}(x)}{2h_{0}\left( x \right)}$ | (S4) |
| --- | --- | --- |

The accurate volume of each section should be calculated as: $V=A\cdot L$; however, as $A$ changes along $x$-axis, such a simple equation no longer applies. Therefore, we divided the length of each section is $n$ discreet quantities ($dL=100 \mu l$) that are small enough so to assume the cross section to be constant on each $dL$, and we computed the volume as:

|  | $V= \sum_{i=0}^{n} A_{i}\cdot dL$ | (S5) |
| --- | --- | --- |

Finally, we calculated the start-up time in each section ($t_{section}=V_{section}\cdot Q_{section}^{-1}$) and the total start-up time ($T$) as:

|  | $T=t_{inlet}+t_{branch}+t_{conduit}$  $\therefore T=\frac{V_{inlet}}{q_{inlet}}+\frac{V_{branch}}{q_{branch}}+\frac{V_{conduit}}{Q_{conduit}}$ | (S6) |
| --- | --- | --- |

where $q_{inlet}=q_{branch}=\frac{Q_{conduit}}{2}$. For the circuit geometry used in this manuscript and $Q_{conduit}=5 \mu l\cdot h^{-1}$, the start-up time computed with Eq. S6 ($\sim1.5 h$) perfectly aligns with experimental results obtained in Supplementary figure 3.

Supplementary figure 4C plots start-up time predictions for circuits with different inlets, $a_{conduit}$ and $a_{branch}$ are kept constant (1 mm and 0.5 mm respectively) while $a_{inlet}$ varies between 0.5 mm and 2 mm. Supplementary figure 4D shows start-up times for the different flow rates tested in this manuscript.


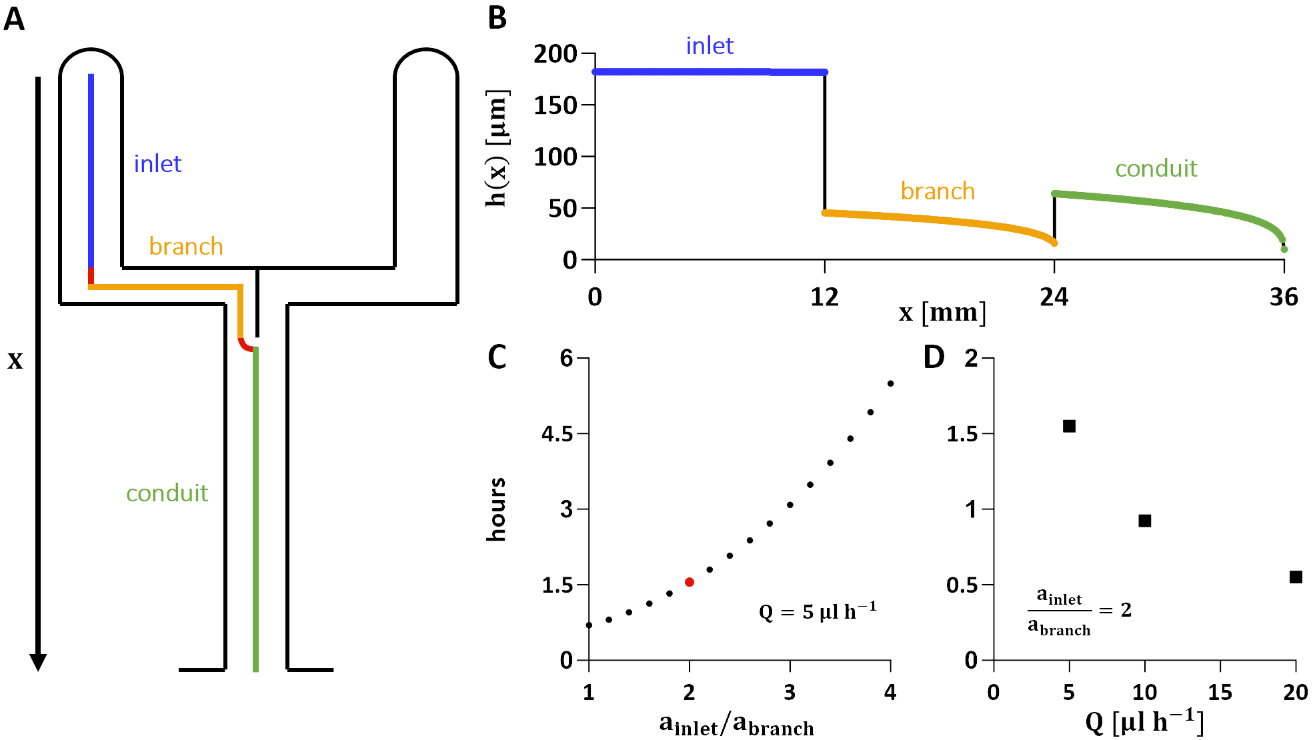


**Supplementary Figure 4. Start-up time approximation.**

**(A)** Schematic of the circuit used. Black lines represent the edges of the conduits while coloured lines indicate approximate location of the central height of the fluid walls in the three sections (inlet-blue, branch-yellow, conduit-green).

**(B)** Plot representing the central height of fluid walls in the three sections (calculated using Eq. S1); $a_{inlet}=1 mm$ and $L_{inlet}=12 mm$, $a_{branch}=0.5 mm$ and $L_{branch}=12 mm$, $a_{conduit}=1 mm$ and $L_{conduit}=12 mm$, $q_{inlet}=q_{branch}=2.5 \mu l\cdot h^{-1}$, $Q_{conduit}=5 \mu l\cdot h^{-1}$.

**(C)** Charts representing estimation of start-up times for different inlet-branch geometries (red dot indicate the geometry used in the paper), and different flow rates.


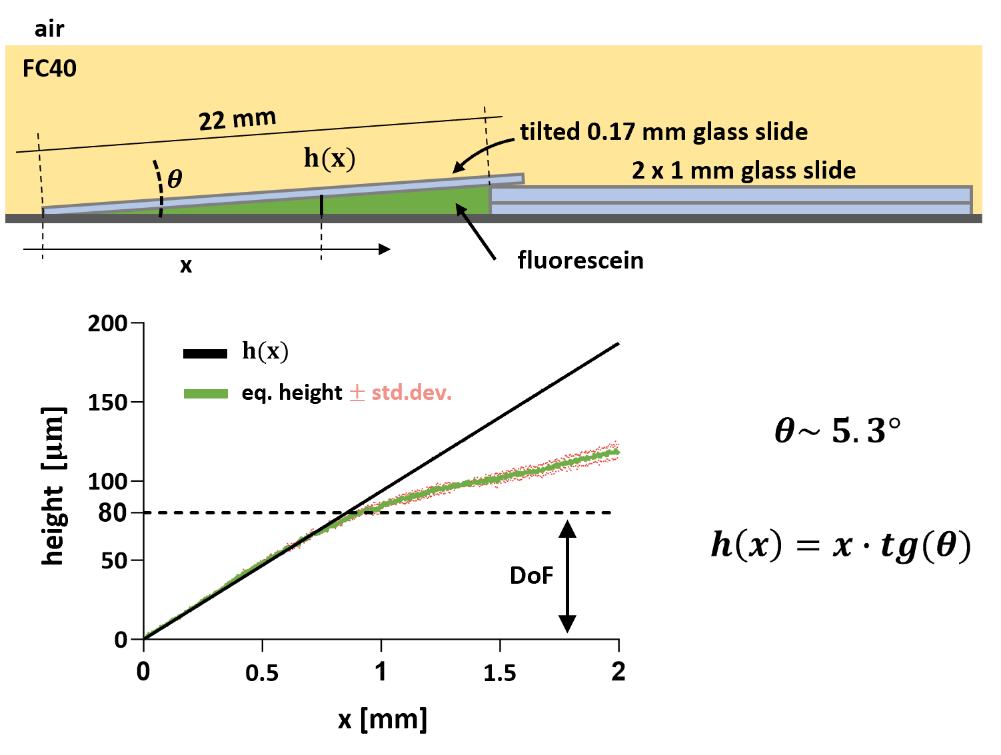


**Supplementary Figure 5. Correlation between fluorescent intensities and heights.**

**(A)** Schematic of the experimental set-up used to prove the linear relationship between recorded intensities and heights. A thin (0.17 mm) glass slide is placed in a standard Petri dish with one edge laying on the dish while the other edge laying on a stack of two 1mm-thick glass slides. The wedge-shaped space underneath the tilted glass slide is filled with a fluorescein solution (see ‘Reagents’ paragraph in the Materials and Methods section), and the dish is then filled with FC40 so to cover the entire structure. This generates a linear height profile that can be easily calculated using trigonometric equations.

**(B)** The intensity profile of the fluorescein is than recorded and converted in equivalent heights multiplying each pixel intensity by the calibration factor (0.79). Within the DoF, the resulting profile (green line) perfectly matches the height profile of the tilted glass slide (black line) demonstrating the accuracy of this measurement technique. As expected, once the DoF is reached the two profiles diverge with the experimental profile being outperformed by the real profile. Pink lines show standard deviation of the measurements (n = 3).


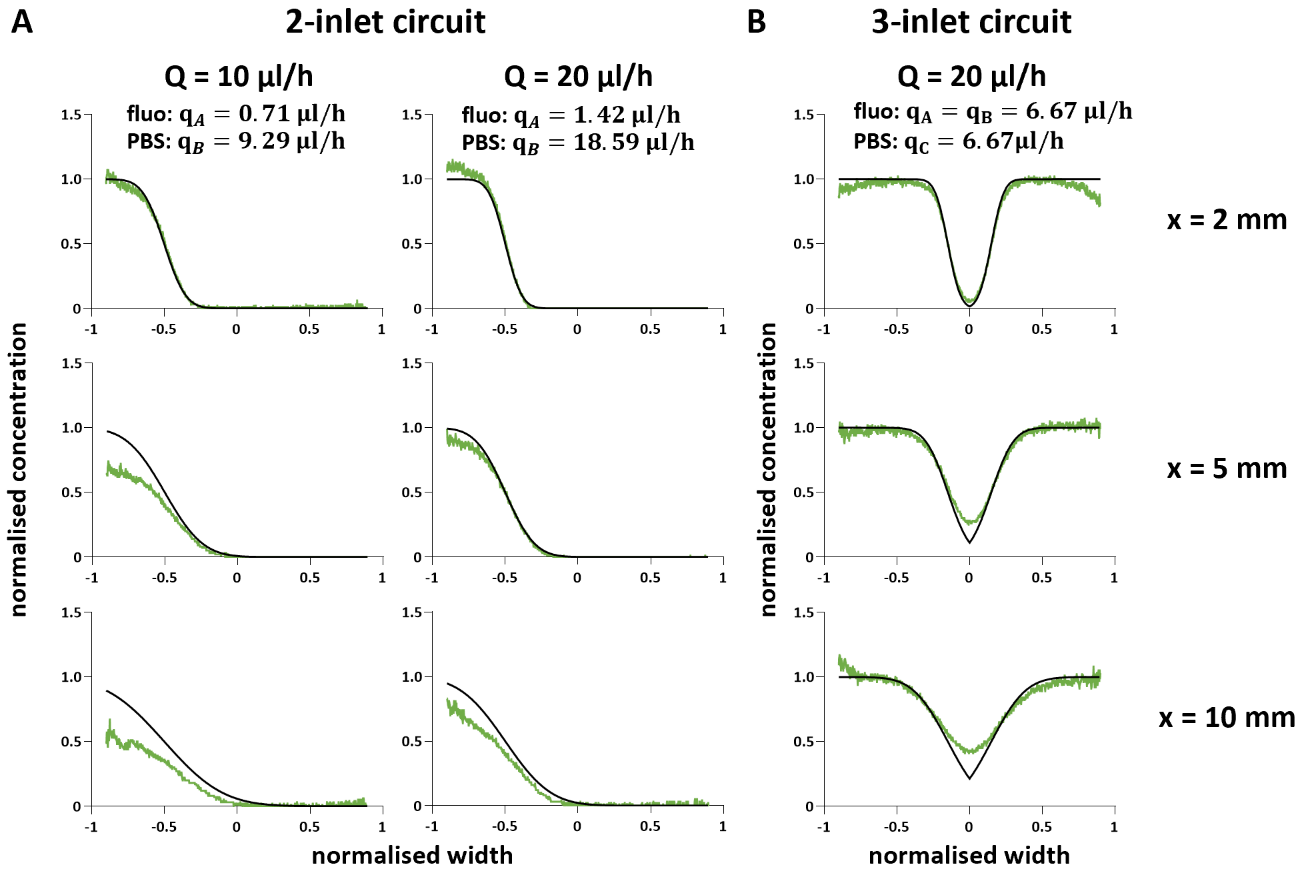


**Supplementary Figure 6. Diffusion gradients resulting from off-centre contact planes.**

**(A)** Y-shaped circuits with $q_{A}<q_{B}$. Conditions and presentation as in Figure 5, with the differences indicated. Eq. 7 (black line) predicts the diffusion gradient seen experimentally (green) when x = 2 mm, but performs progressively less well with distance from the junction. As $s_{1}= -0.5$, velocity of the contact plane lowers (Fig. 5B) and it becomes comparable to diffusion velocity ($Fo\sim1)$; therefore, the fluorescein stream concentration falls below $C_{0}$ (and so it can no longer be considered to be diffusing from an infinitely large reservoir). Consequently, the green trace lies below the black line.

**(B)** Trident. Conditions and presentation as in Figure 6 where fluorescein streams flow on either side of a central PBS stream. Eq. 7 (black line) again predicts the diffusion gradient seen experimentally (green) when x = 2 mm, but performs progressively less well with distance from the junction. Since the contact planes are close to the centre of the conduit ($s_{1}= -0.15, s_{2}=0.15$), the volume of PBS in the centre is small, and so the concentration of fluorescein in the PBS stream can no longer be considered as zero. Consequently, the green trace now lies above the black line at position 0.


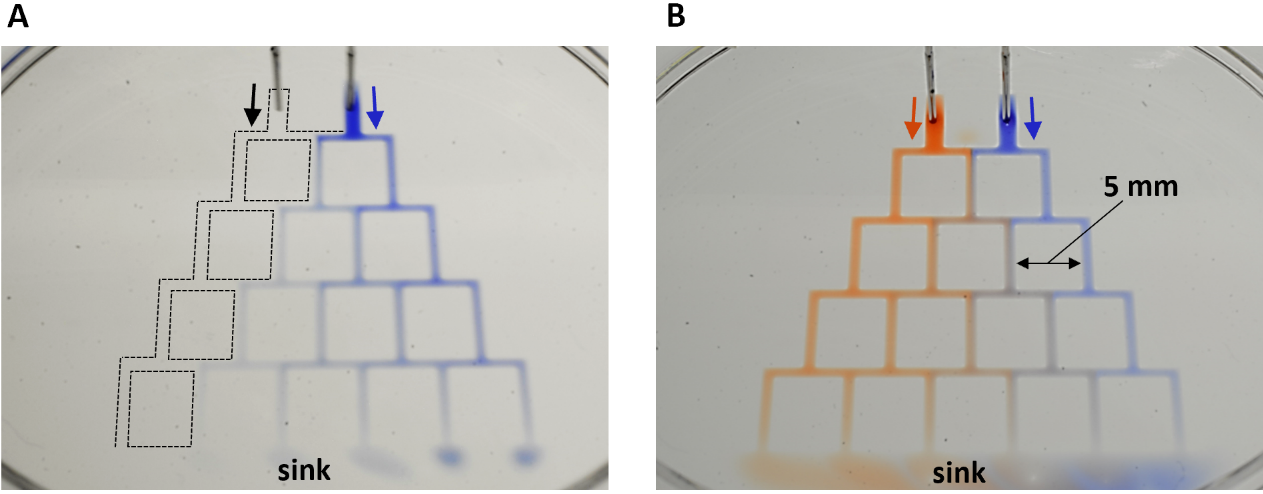


**Supplementary Figure 7. Concentration gradients formed using Christmas-tree circuits with fluid walls in a 60 mm Petri dish**

**(A)** PBS is infused in the left inlet while PBS + blue dye (resazurin 0.4mg/ml) is infused in the right one. Dashed lines show the position of branches where only PBS is flowing.

**(B)** PBS + red dye (Allura red 4 mg/ml) is infused in the left inlet while PBS + blue dye (resazurin 0.4 mg/ml) is infused in the right one. The colour gradient is visible in the common sink
